# Novel insights into RabA2b intracellular localization suggest a role in plasmodesmatal function

**DOI:** 10.3389/fpls.2025.1605468

**Published:** 2025-10-09

**Authors:** Vivek Ambastha, Dafna Tidhar, Ifat Matityahu, Yehoram Leshem

**Affiliations:** ^1^ Department of Plant Sciences, MIGAL – Galilee Research Institute, Kiryat-Shmona, Israel; ^2^ Faculty of Sciences and Technology, Tel Hai College, Upper Galilee, Israel

**Keywords:** plasmodesmata, Rab small GTPases, RabA2b, plasma membrane trafficking, microchannel transport, Hechtian strands, water stress

## Abstract

Plasmodesmata (PD) are microchannels that bridge together neighboring plant cells by connecting their plasma membranes to each other. Thus, the PD establish the symplast, a cytoplasmic continuum that allows free transport of water and other small molecules between cells. Also, PD communicate with the cytoplasm *via* intercellular trafficking. Rab small GTPases are key regulators of vesicle trafficking, though surprisingly, their role in PD targeting and trafficking is still obscure. Here we show, for the first time, that the RabA member, RabA2b, associates with the PD. Furthermore, its overexpression in Arabidopsis facilitates symplastic mediated transport. Additionally, during osmotic stress, the presence of RabA2b was observed along the Hechtian Strands (HS) as well as at HS-cell wall docking sites. Our findings provide new insights into the potential involvement of RabA2b in PD permeability regulation and mediation of the symplast integrity, necessary for water maintenance during water stresses.

## Introduction

Plasmodesmata (PD) are microchannels that cross the cell walls of neighboring plant cells bridging their cytoplasms to each other. The cytoplasmic continuum establishes the symplastic pathway that allows water transport between cells and tissues without the need to cross the plasma membrane (PM) semipermeable barrier. The PD is structured by a sleeve that fuses the PMs of neighboring cells, and merges their cytoplasms. The sleeve harbors a desmotubule composed of endoplasmic reticulum (ER) protrusions of both cells, which fills the sleeve’s interior and connects both cell’s ER systems to each other (Sager and Lee, 2018; Faulkner, 2018). The PD connection with the endo-membrane system generates multiple paths for communication with the cytoplasm through intercellular trafficking (Li et al., 2021). Additionally, the PD allows the passage of many small molecules (<1kDa) that diffuse freely between neighboring cells. Other molecules, larger in size than the channel pore diameter, can actively pass through these channels via advanced and highly regulated transport mechanisms (Sager and Lee, 2018; Faulkner, 2018). The molecules transported via the PD can play a role in primary and secondary metabolisms as well as in cell-to-cell communication and signaling (Miras et al., 2022; Petit et al., 2020). The PD are essential for proper plant growth and development (Burch-smith et al., 2011) and are highly involved in response to biotic stresses (Cheval and Faulkner, 2018; Liu et al., 2021; German et al., 2023).

Various membrane proteins, including single- and multipass integral transmembrane proteins have been identified to target the plasmodesmata. These proteins, such as callose synthases, cell surface receptors, and the family of plasmodesmata-located proteins (PDLPs), play key roles in plasmodesmal permeability regulation (Cui and Lee, 2016; Rosas-Diaz et al., 2018; Luna et al., 2023). Additional proteins that are known to target plasmodesmata include membrane tethering or cytoskeletal regulating components (Brault et al., 2019; Diao et al., 2018). Despite the key roles of the membrane proteins in PD functioning, their mode of delivery to the PD is still unknown.

Rab small GTPases are key regulators of vesicle trafficking (Nielsen, 2020). The Rab super family in Arabidopsis consists of 57 members that are divided into 8 sub-families (A-H). The different Rabs are localized to distinct intracellular membranes and mediate the multiple steps along the exocytic and endocytic pathways. RabA is the largest Rab subfamily, consisting of 26 members, and was shown to be involved in PM trafficking and cell wall (CW) metabolism (Lycett, 2008; Lunn et al., 2013). Surprisingly, their link to PD targeting and trafficking is still obscure. Recently, several Rab members were identified in proteomes of plasmodesmata enriched fractions (PEF) in Poplus (Leijon et al., 2018) and Arabidopsis (Li et al., 2024). Nevertheless, the PD localization of these Rabs was not validated.

Recently, we reported that RabA2b expression was strongly upregulated in Arabidopsis vasculature during osmotic stress in an ABA dependent manner (Ambastha et al., 2021). We also showed that overexpressing RabA2b in Arabidopsis altered the PM proteome and improved the plant’s drought tolerance. To determine whether RabA2b reached the PM, we fused it with GFP and tested for its colocalization with the PM marker PIP2A, in double transgenic Pro35S:GFP-RabA2b/Pro35S:PIP2A-mCherry plants. In the sub-cellular localization study, conducted by confocal microscopy, we detected in mature shoot and root cells substantial overlap between the green (RabA2b) and red (PIP2A) fluorescent signals (Ambastha et al., 2021).

Here we closely studied the features of RabA2b distribution on the PM and showed for the first time, that RabA2b, associates with the PD, affecting symplastic mediated transport. Apparently, no RabA protein has been previously reported to be associated with the PD, thus, we open new research avenues regarding the association of these trafficking proteins with the PD.

## Methods

### Plant materials and growth conditions

The stable single or double transgenic Arabidopsis plants expressing RabA2b:GFP or both RabA2b:GFP and PIP2A:mCherry under constitutive promoter in wild type (WT, Col-0 background), respectively, we described previously (Ambastha et al., 2021), were used for the co-localization studies. Seed surface sterilization and plating was performed as we described previously (Ambastha et al., 2020).

### Osmotic shock and plasmolysis

Six-day-old seedlings were placed on a glass slide, and 100µl of 750mM sucrose solution was added directly over it. The slide was incubated for 5 minutes at room temperature (RT) and observed under the 63X oil objective of an LSM confocal microscope for GFP and mCherry-specific fluorescence as described below in “Confocal microscopy” section.

### Plasmodesmata callose staining

The Aniline blue-based PD localized Callose staining and comparative quantification were performed as described by Raul and Epel (Zavaliev and Epel, 2015). In brief, the 6-day-old Arabidopsis leaves of the mentioned single or double transgenic lines were fixed in 96% ethanol at RT for 3 hours. Then rehydrated with DDW for one hour following incubation in aniline blue solution (0.01% aniline blue in 0.01M K_3_PO_4_) for 45 minutes at RT. Imaging was performed by confocal microscope as described below in “Confocal microscopy” section.

### Symplastic dye movement assay

The symplast tracer 8-hydroxypyrene-1,3,6-trisulfonic acid (HPTS) was used for studying symplasmic movement in RabA2b overexpression (OE) lines which was performed according to Ritesh et al (Ritesh et al., 2016). Briefly, a 1% agarose gel was prepared and supplemented with HPTS. Three day-old etiolated seedlings were excised at the base of the hook and positioned on a cover slide to ensure specific contact between the hypocotyl and the HPTS agarose block. The seedlings were examined under a confocal microscope following a 30-minute loading period and a subsequent 10-minute washing step. The extent of dye movement from the loading point was compared between two independent RabA2b overexpressing lines (OE 6.4 and OE 11.4) and wild type (Col-0) seedlings. HPTS fluorescence was determined in the hypocotyl by a confocal microscope 15 minutes after dye washing.

### Confocal microscopy and image analysis

**Figure 1:**
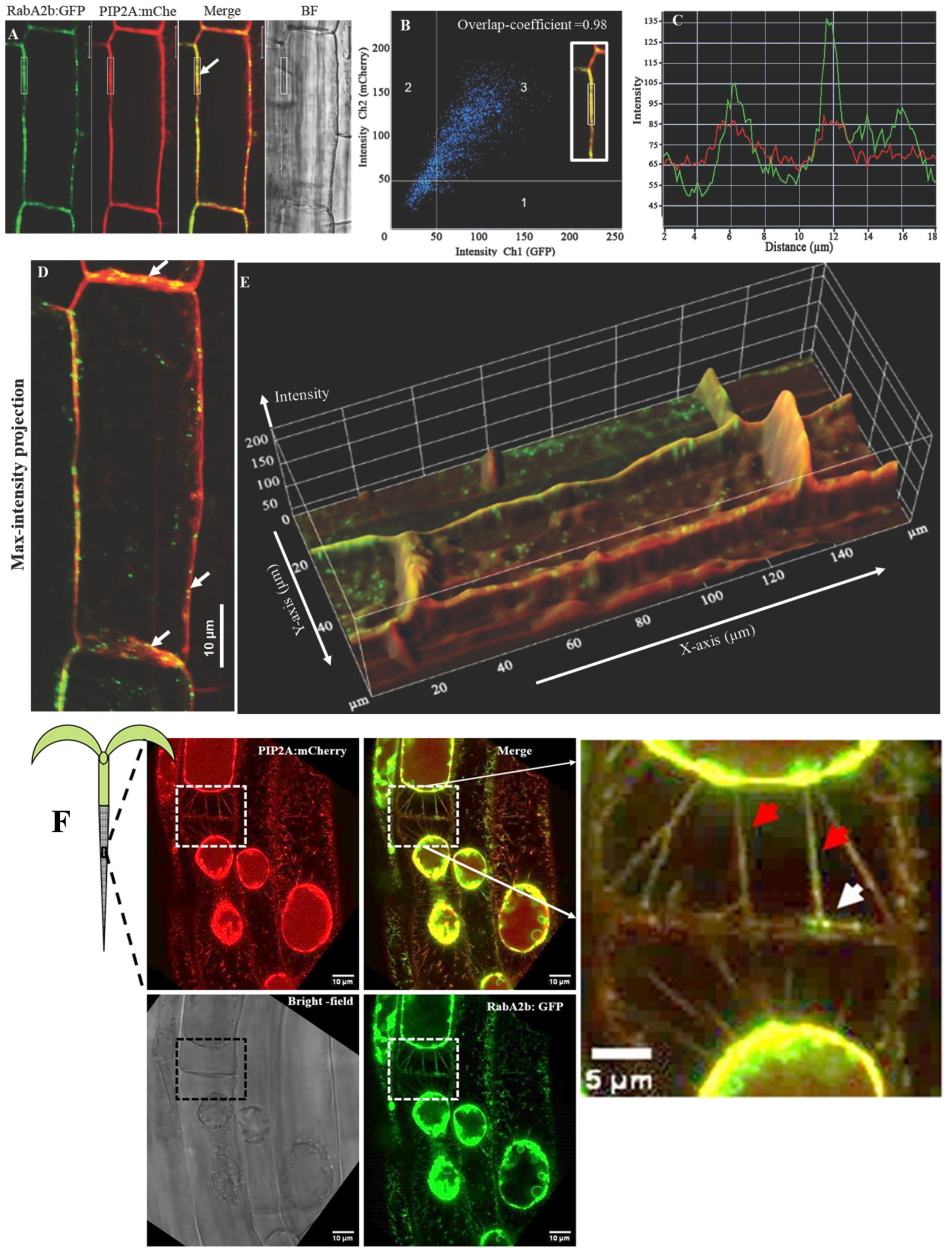
Spatial distribution of RabA2b:GFP in Arabidopsis root cells during standard and osmotic stress conditions. Representative confocal images of a double transgenic line expressing 35S:RabA2b:GFP and the plasma membrane marker aquaporin PIP2A:mCherry showing spatial distribution of RabA2b. **(A)** The 2D split image shows elongated root cells in GFP, mCherry, Merge, and bright-field (BF) filters. The small rectangular box represents the region of interest (ROI) selected for colocalization analysis and intensity profiling of GFP and mCherry fluorescence. **(B)** The colocalization analysis performed on ROI (in inset) is presented as a scattered plot divided into four quadrants. The third quadrant represents the overlapping pixel with significantly high fluorescence in both GFP and mCherry filters having an overlapping coefficient equal to 0.98 (calculated by Zen blue). **(C)** The profile graph of ROI in line-scans mode shows two separated intensity peaks of GFP fluorescence while only minor fluctuation in the mCherry intensity indicating the presence of random pockets of RabA2b: GFP on the plasma membrane. **(D)** The merged image of the maximum intensity projection of GFP and mCherry was obtained from Z-stack. The white arrow in the longitudinal and transverse axis of the cell membrane marks the presence of a punctate structure. Also, these GFP-labeled punctate structures are uniformly distributed in the cytoplasm. **(E)** The 2.5D intensity projection is obtained by processing the 3D merge of GFP and mCherry filters, where the fluorescence pixel intensity is represented by peak extension in Z- the direction. The pixel with dual fluorescence is colored yellow with equal extension in Z-direction. The X and Y-axis represent the length and breadth of the cell respectively. **(F)** Intracellular localization of RabA2b during osmotic stress in Arabidopsis root cells. Representative fluorescent confocal microscopy images of plasmolyzed root cells from a 6-day-old Arabidopsis plant expressing mCherry: PIP2A and GFP: RabA2b. Red arrows indicates Hechtian Strands localization of RabA2b while white arrow point to Hechtian Strands – cell wall docking site with RabA2b localization. The complete Z-stacks of the presented cells can be viewed in **Supplementary Figure 2**.

A Zeiss LSM 700 laser scanning confocal microscope (LSCM) was used in this study. The aniline blue-stained Arabidopsis leaves were observed using a plan-Apochromat 20 ×/0.8 M27 objective. The laser setting for CFP and Aniline blue was 405 nm, while for GFP (RabA2b) and mCherry (PIP2A), the laser setting was 488 nm, and 555nm was used to capture the images. Due to AB wide emission spectra (that can “bleed” into the GFP channel), the fluorescent signals were sequentially scanned. The laser intensity, digital offset, master gain, and pinhole were kept constant during all experiments. Zen black software (Carl Zeiss) was used for Co-localization analysis, intensity profiling, and maximum intensity projection image rendering. Fiji software’s 3D surface plot tool (https://imagej.net/software/fiji/) was used to generate the 2.5 D intensity projection (**Figure 1E**). 2.5D image spreads out the intensity of the 2D image in the Z-plane, visualizing the GFP or mCherry fluorescence intensity of the x and y plane as a high or low peak area projecting on the Z-axis (Seema et al., 2021). The GFP and mCherry fluorescence were also detected in mature-elongated root cells and hypocotyl cells of the double transgenic line using similar microscope settings. In addition, the RabA2b:GFP signal was also determined using the mentioned confocal settings in young root cells (from the dividing zone) of Arabidopsis plants expressing RabA2b:GFP which were incubated for 15 minutes with 2µM of the specific endo-membrane tracer FM 4-64 (Invitrogen, T3166) which was excited at 555nm as we described previously (Leshem et al., 2007; Ambastha et al., 2021). For HPTS visualization, the hypocotyl region was focused using a 20X objective lens of the above-mentioned microscopeusing an excitation laser of 488nm and emission between 500 and 550 nm. The sample was scanned under tiles mode to capture the complete length of the hypocotyls for tracking the dye movement (Ritesh et al., 2016) using ImageJ software. The green fluorescence of HPTS was measured from the cut end of the hypocotyl in the RabA2b overexpressing lines (OE#6.4 and OE%11.4) and wt Col-0 background.

## Results

### Spatial distribution of RabA2b on PM ascertained by confocal microscopy

We performed a deep analysis of our previously published result of RabA2b PM localization in elongated mature root cells (Ambastha et al., 2021) and noted a high overlapping coefficient (= 0.98), between the RabA2b:GFP and the PM marker PIP2A-mCherry signals, indicating the trafficking of RabA2b to the PM (**Figures 1A** and B). To better understand the spatial distribution pattern of RabA2b, we performed intensity-profiling analysis, by applying a line scan to the overlapping regions, which provides a graphical display of distance versus fluorescence intensity along the selected area (**Figure 1C**). This analysis enabled us to observe peaks of high GFP intensity along the membrane, while the mCherry intensity appeared to be approximately constant along the same line scan (**Figure 1C**). Therefore, while PIP2A:mCherry distributes more uniformly on the PM, RabA2b::GFP is concentrated at specific spots along the PM. Further transformation of the full z-stack projection image (**Figure 1D**) into 2.5D configuration (**Figure 1E**) confirmed the presence of intense GFP spots in the cytoplasm as well as on the PM throughout the cell’s periphery. Previously, GFP spots have been shown in the cytoplasm to represent FM 4–64 labeled endosomes (Ambastha et al., 2021). However, considering our observations along the PM, we speculated that the punctate GFP distribution reflects plasmodesmatal localization of RabA2b. Interestingly, the mean RabA2b:GFP signal intensity of the elongated root cells was higher in transverse PM regions as compared with longitudinal PM regions. (**Supplementary Figure 1A**). Since the transverse PM regions of these cells reflect positions where the cell plate has matured into a complete PM, and since several RabA proteins including members of the RabA2 subfamily were observed to localize during cytokinesis on the cell plate (Chow et al., 2008; Berson et al., 2014; Davis et al., 2016), we further examined the RabA2b intracellular localization in younger dividing root cells. We observed strong GFP signal in the forming cell plate of the dividing cells (**Supplementary Figure 1B**). Upon completion of cytokinesis, the strong GFP signal was observed on the leading edges of the complete plate/newly formed PM (**Supplementary Figure 1B**). As primary plasmodesmata are formed at the cell plate (Knox et al., 2015), RabA2b presence at that pre-PM organ suggests its possible involvement in primary plasmodesmata formation as well as in cytokinesis.

### Localization of RabA2b during osmotic shock

During severe plasmolysis, the protoplast shrinks and retracts from the CW, and the strands of the PM (named Hechtian strands – HS) remain docked to the CW at the PD or other binding sites and are hence stretched (Oparka, 1994; Lang et al., 2014; Arico et al., 2023). Thus, our next experiment was to subject our double transgenic plants to osmotic shock (750mM sucrose for 5 minutes), in attempt to observe the HS docking sites. We examined by confocal microscopy the distribution of the red (PIP2A:mCherry) and green (RabA2b:GFP) fluorescent signals in cells from the root hair zone. The inspected cells exhibited convex plasmolysis in which the protoplast volume shrank significantly (**Figure 1F**). The PM retained its connections with the CW through multiple HS, that were visible in the red fluorescent filter (**Figure 1F**). The RabA2b:GFP signal was observed in HS–CW docking sites, which supposedly indicate PD sites (white arrow in inset image in **Figure 1F**, and in other HS-CW docking positions along the full z-stack presented in **Supplementary Figure 2**). Intriguingly, the GFP signal representing RabA2b localization was also elegantly aligned with the entire HS (**Figure 1F**, red arrows in inset image).

### RabA2b co-localization with PD staining

**Figure 2:**
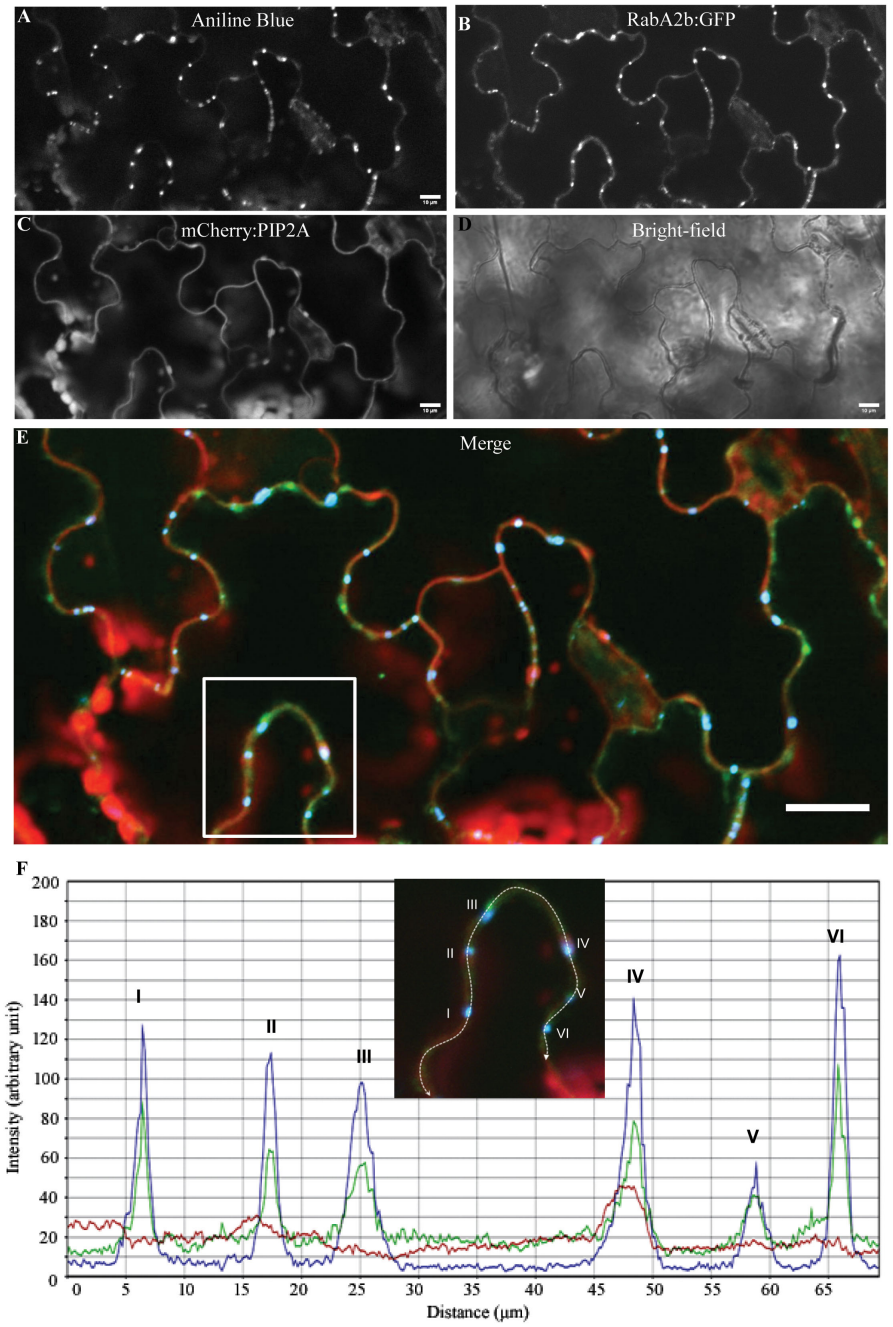
Distribution of RabA2b on the plasma membrane and its colocalization with callose depositions. **(A–F)** Representative confocal images of Aniline blue (AB) staining of double transgenic Arabidopsis plants expressing RabA2b:GFP and PIP2A:mCherry under 35S promoter. **(A)** Aniline blue (AB) fluorescence from membranal callose depositions in rosette leaf epidermal cells. The leaves were fixed with ethanol prior to AB staining as described in the methods. **(B)** GFP fluorescence of RabA2B. **(C)** mCherry fluorescence of the plasma membrane marker PIP2A. **(D)** Enlarged merged image of the images presented in A-C (AB fluorescence with GFP and mCherry) showing colocalization of all the three signals in the same spot. **(E, F)** The intensity plot of the area marked by the white box marked in **(D)** showing intensity peaks of GFP and Aniline blue overlapping with each other, while the intensity of mCherry is constant through the selected area. Red Roman numbers in F correspond to the white Roman numbers in **(E)** Scale bar in D = 20µm.

Since callose is known to be present in large quantities in the PD, aniline blue (AB) staining which binds to callose and produces blue fluorescent signal, is commonly used to visualize PD (Zavaliev and Epel, 2015). Therefore, we stained rosette leaves of the double transgenic plants mentioned above with AB. The confocal microscopy analysis which followed allowed in leaf epidermal cells the observation of three fluorescent signals: red (PIP2A:mCherry), green (RabA2b:GFP), and Blue (Callose). (**Figures 2A-C**). Merging the three fluorescent channels showed clearly that the green and the blue punctate signals completely overlapped on the PM that was marked by red signal (**Figure 2E**). Further intensity-profiling analysis that was performed on the area selected in **Figure 2D** (which was enlarged in **Figure 2E**), showed that the green and blue, fluorescent intensities completely overlapped at six blue/green punctate sites (**Figure 2F**). At each appearance of the blue fluorescence, an overlapping green fluorescence was observed. In addition, the mCherry signal was constant throughout the line selected for fluorescence intensity profiling, indicating the uniform distribution of PIP2A:mCherry on PM. The AB staining results therefore suggest that RabA2b associates with the PD. To assess whether RabA2b overexpression has affected the PD abundance, we quantified the PD number in AB stained leaf epidermal cells of the transgenic lines and wt. The tested genotypes exhibited similar number of PDs per area unit (**Supplementary Figure 3**).

### Effect of RabA2b overexpression on PD permeability

**Figure 3:**
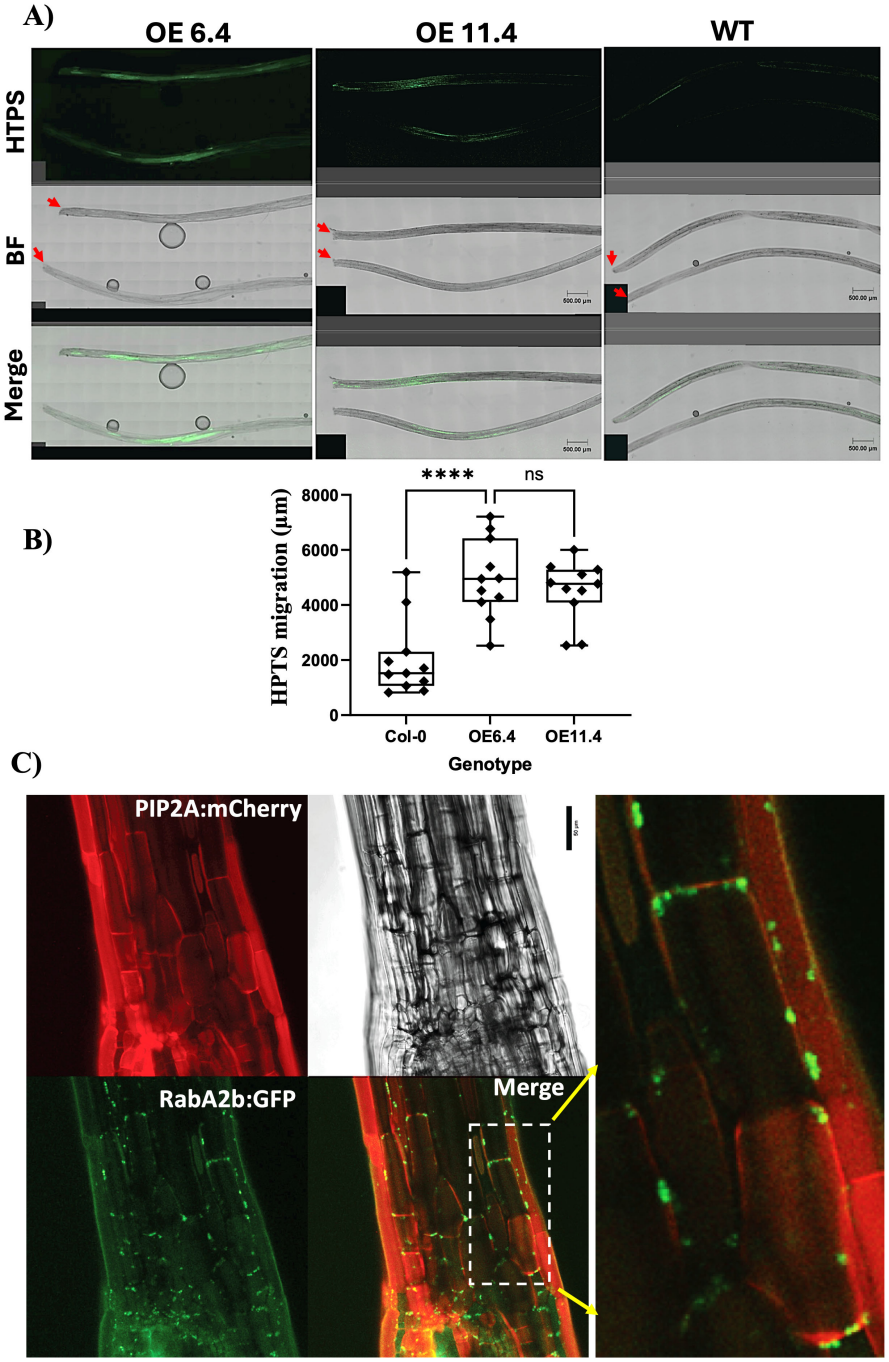
The effect of RabA2b overexpression on symplastic HPTS movement and RabA2b PM distribution in hypocotyl cells **(A)** Representative fluorescent images of HPTS migration assay in hypocotyls of wt and two independent lines of RabA2b overexpressing plants (OE 6.4 and OE 11.4). The hypocotyls were excised below the cotyledons of young, etiolated seedlings. Red arrows indicate the point of HPTS loading following Kumar et al, 2016. HPTS fluorescence was determined by confocal microscope, as described in methods. **(B)** Quantification of the HPTS migration distance (presented in micrometers) in the tested genotypes in several independent assays (n= 11 hypocotyls per genotype, ± SE). Asterisks indicated statistical difference at p <0.0001 as determined by a two-tailed unpaired t-test, ns – not significant. Scale bar in A = 500 μm, BR=Bright Field. **(C)** Representative confocal images of hypocotyl cells in the double transgenic line expressing 35S:RabA2b:GFP and the plasma membrane marker aquaporin PIP2A:mCherry (presented above in **Figure 1**). Provided are images of the separate red (PIP2A:mCherry) and green (RabA2b:GFP) filters, merged and bright field. The image on the right side is enlargement of the white boxed area in the merged image. Scale bar in C = 50 μm. Note the punctate appearance of the GFP signal (RabA2b) and its colocalization with the PM red signal.

Next, to determine whether RabA2b localization at the PD affects the PD permeability in the transgenic plants, we used the small (0.5 kDa) symplastic fluorescent tracer 8-hydroxypyrene-1, 3, 6-trisulfonic acid (HPTS). HPTS is membrane-impermeable and therefore moves between cells *via* the PD (Kim et al., 2002; Xu et al., 2012; Shih et al., 2019). The HPTS fluorescence was determined in the tested genotypes by confocal microscope 15 minutes after dye washing, and the dye migration distance from the loading point was measured. The HPTS migrated significantly longer distance in the RabA2b overexpressing lines as compared to wt (**Figures 3A, B**). Therefore, these results indicate that the PD permeability was increased and thus facilitated by RabA2b. To validate the presence of RabA2b at the PM of hypocotyl cells, we studied by confocal microscope the double transgenic line expressing 35S:RabA2b:GFP and the plasma membrane marker aquaporin PIP2A:mCherry. The RabA2b:GFP signal distributed in the hypocotyl cells in punctuated manner (as observed in the root and leaf cell type mentioned earlier), and overlapped with the PIP2A:mCherry signal, indicting its co-localization with the PM (**Figure 3C**).

## Discussion

Since the discovery of plant RabA small GTPases, the intracellular localization of several RabA members has been well characterized (Rutherford and Moore, 2002; Nielsen, 2020). Nevertheless, the PM punctuation patterning we report here for RabA2b was not demonstrated so far in any of the other mentioned RabA isoforms. Considering that the PD is structurally integrated with the PM as well as with the cell’s internal membrane system, the PM trafficking proteins can be expected to associate with the PD. Indeed, we show here that RabA2b colocalizes with the classic PD marker AB (**Figure 2**). Members of the intracellular trafficking machinery, including several Rab small GTPases, have been recently identified in PD enriched proteomes (Leijon et al., 2018; Li et al., 2024). Yet, to our knowledge, the study we present here is the first to demonstrate the colocalization of any Rab member with the PD.

Recently, RabA2b was identified in the PD proteome reported by Li et al (Li et al., 2024), a result which chimes with our colocalization observations of RabA2b at the PD site. Furthermore, this proteomic data captured the interactors of PDLP5 and PDLP6 using the proximity labeling technique and identified RabA2b as an interactor of PDLP5, which is known to be involved in regulation of PD permeability (Lee et al., 2011). Thus, it is possible that the increased HPTS symplastic transport we observed in the RabA2b overexpressing plants (**Figures 3A-B**) results from direct interaction of RabA2b with PDLP5. Alternatively, based on RabA2b involvement in CW metabolism (Lycett, 2008; Lunn et al., 2013) it can act by delivering to the PD other cargos which can be essential for its functionality, such as cell wall materials or cell wall processing enzymes, which can affect PD permeability. Future high-resolution studies of the PD structure may reveal differences between the tested genotypes.

During osmotic stress, the observation of RabA2b at the HS-CW docking sites as well as along the HS provide further support for RabA2b association with the PD, and additionally, possible involvement in symplast maintenance under water-limiting conditions. During the severe plasmolysis, the stretched HS can cause localized high-tension conditions between the HS and its connecting points with the PD (and the other CW binding sites). To withstand this tension the plasticity of the HS as well as the PD and the other anchored positions, needs to be locally altered/adjusted and, if damaged, repaired or recycled. Failure to adapt to increased plasmolysis conditions can eventually lead to the disintegration of the symplast route (Quiles et al., 2003), which is vital for water relations at the cellular as well as whole plant levels (Spicer, 2014). Since the mammalian homologue of RabA proteins, Rab11 proteins, are known to be involved in recycling processes (Nielsen, 2020), it is possible that during osmotic stress, RabA2b plays a role in membrane repair of the HS and the PD, allowing the plasticity adjustments required for easing/relaxing the high-tension conditions from the membranes at these sites.

The association of Rab protein with the PD, on one hand, being involved in vesicle trafficking, would be an expected phenomenon, while on the other hand, has not been previously reported. We thus open new avenues of research regarding RabA protein trafficking pathways via the PD. Furthermore, water stresses negatively affect a wide range of crops and are predicted to increase during climate change (Daliakopoulos et al., 2016; Mora et al., 2018). Therefore, the possible involvement of RabA2b in symplast maintenance during water stress conditions is another important phenomenon to be further explored.

## Data Availability

The original contributions presented in the study are included in the article/**Supplementary Material**. Further inquiries can be directed to the corresponding author.

## Supplementary material

Supplementary Figure 1 Quantitative and qualitative distribution of RabA2b PM signals in elongated-mature and young root cells. **(A)** Representative confocal image of elongated root cells from transgenic Arabidopsis plants overexpressing RabA2b:GFP. The yellow rectangle is the transverse membrane, while the purple rectangle represents the longitudinal axis of the cell membrane. **(B)** The mean GFP fluorescence signal quantification per unit length (in microns) at the transverse and longitudinal axes. The GFP signal was measured by ImageJ software using the ROI manager tool. Scale bar=20µm. Eleven root cells of 3 independent transgenic plants were randomly selected, and their transverse and longitudinal PM sections were analyzed. The asterisk represents p value <0.0001 as determined by a two-tailed unpaired t-test. **(C)** Spatial distribution of RabA2b:GFP in young dividing Arabidopsis root cells. Representative confocal images of root tip cells from the division zone of Arabidopsis transgenic plants over expressing RabA2b:GFP. The roots were incubated with the endomembrane marker FM4-64, washed and examined by confocal microscope as we described previously (Ambastha et al., 2021; Leshem et al., 2007). Note the co-localization of RabA2b green fluorescent signal with the evolving cell plate during cytokinesis (upper row), and the leading strands of the complete cell plate/newly formed PM, upon cytokinesis completion (bottom row). Scale bar = 5 µm.

Supplementary Figure 2 Intracellular localization of RabA2b during osmotic stress in Arabidopsis root cells. Representative fluorescent confocal microscopy movies of plasmolyzed root cells from a 6-day-old Arabidopsis plant expressing mCherry: PIP2A and GFP: RabA2b. The videos shows the complete Z stack of the images presented in **Figure 1F**. **(A)** AVI movie of the green (RabA2b) fluorescent filter - Video 1. **(B)** AVI movie of the red (PIP2A) fluorescent filter - Video 2. **(C)** AVI movie of the merged green and red fluorescent signals - Video 3.

Supplementary Figure 3 Quantification of PD number in wt and RabA2b overexpressing plants. **(A)** Representative confocal microscopy images of AB fluorescence in cotyledon pavement cells of wt and transgenic Arabidopsis plants overexpressing RabA2b (OE 6.4 and OE 11.4). AB staining and AB fluorescent detection were performed as described in methods. **(B)** Quantification of PD number in the tested genotypes (per 100µm^2^ area). Scale bar in A = 20µm. In B 14 epidermal cells, respectively, were randomly selected from 3 cotyledons born on 3 plants of the tested genotypes. ns represents no significant difference among means as observed after one-way ANOVA analysis.
