# Supplementary figures and images for: Novel insights into RabA2b intracellular localization suggest a role in plasmodesmatal function
Source: Front Plant Sci. 2025 Oct 9;16:1605468. doi: 10.3389/fpls.2025.1605468 (PMC12573287; doi:10.3389/fpls.2025.1605468)

**A**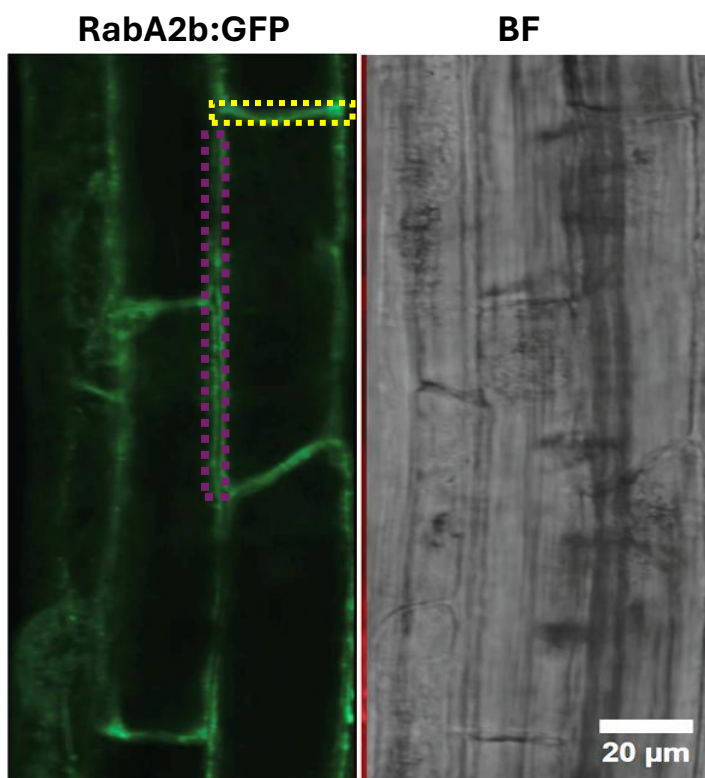**B**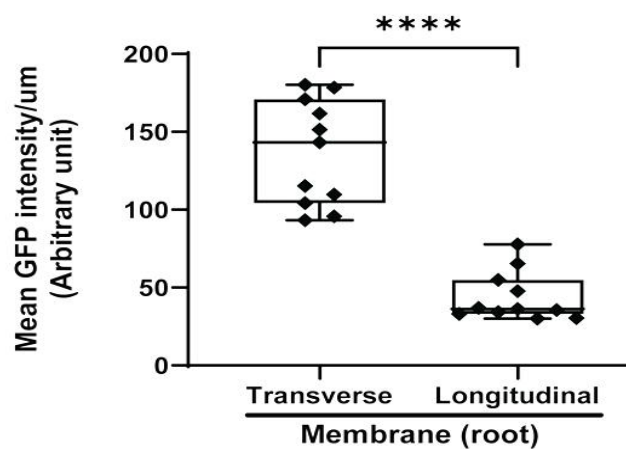**C**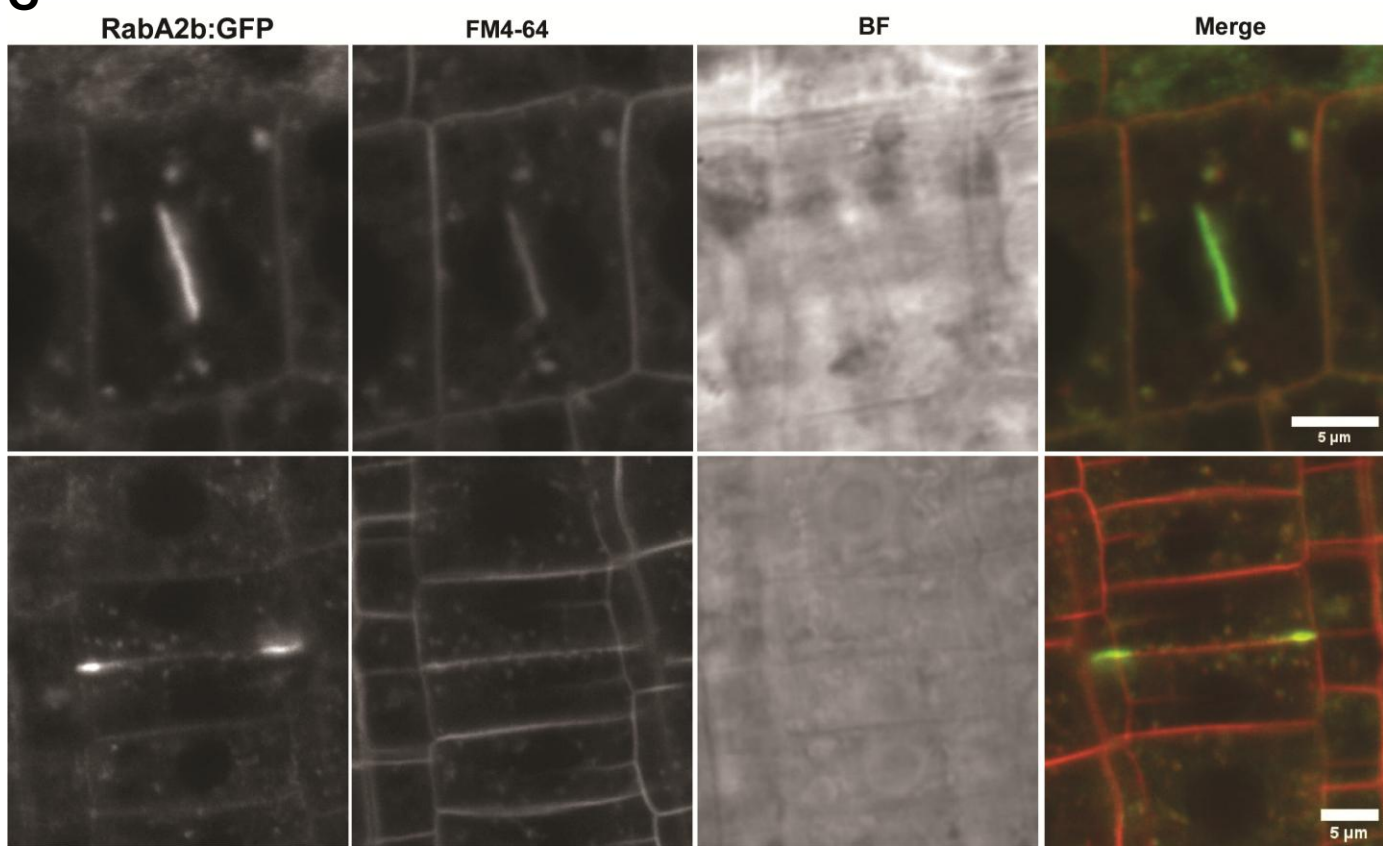

**Supplementary Figure 1**

Supplement: Supplementary file 4 [file DataSheet1.pdf]

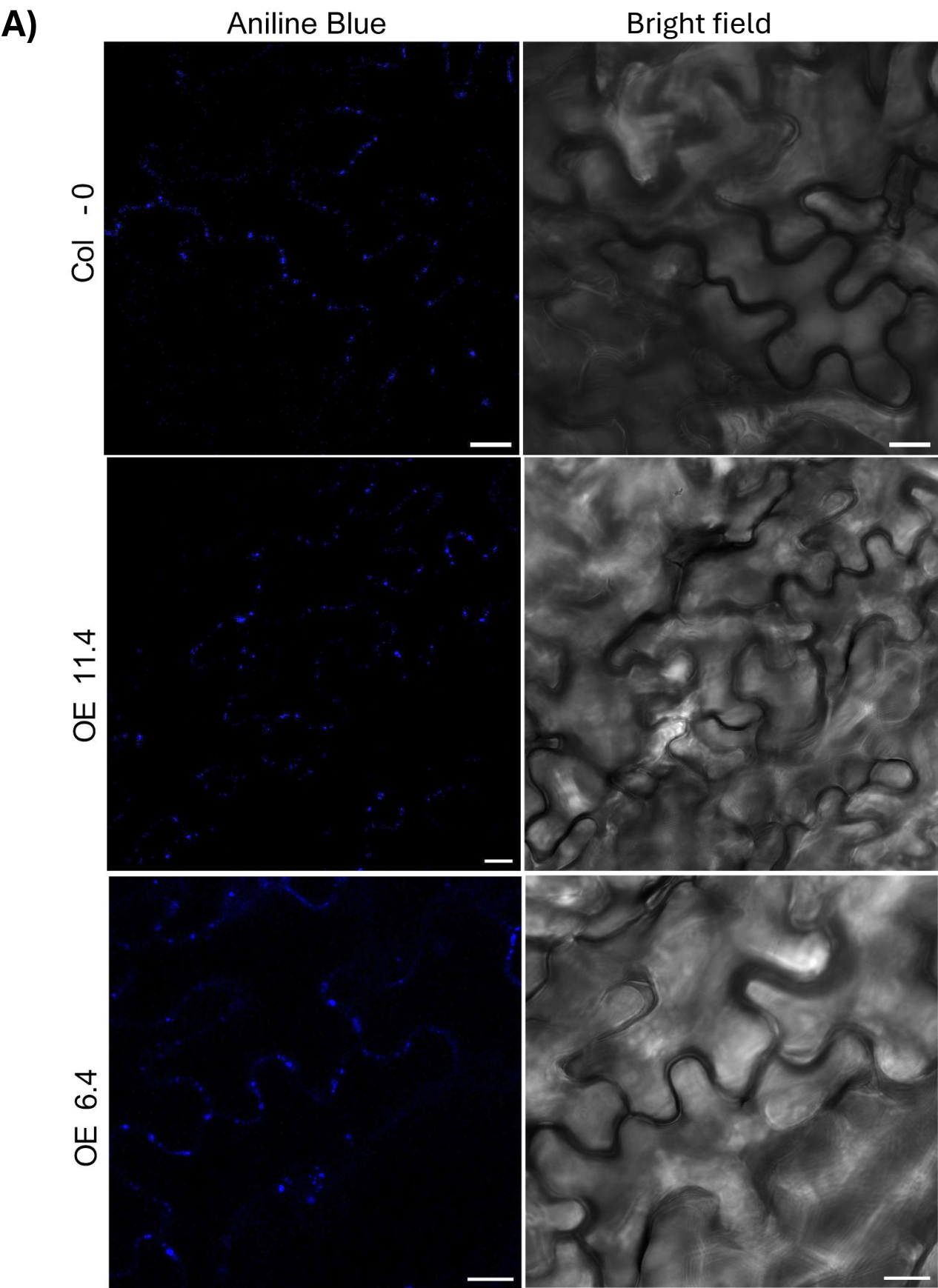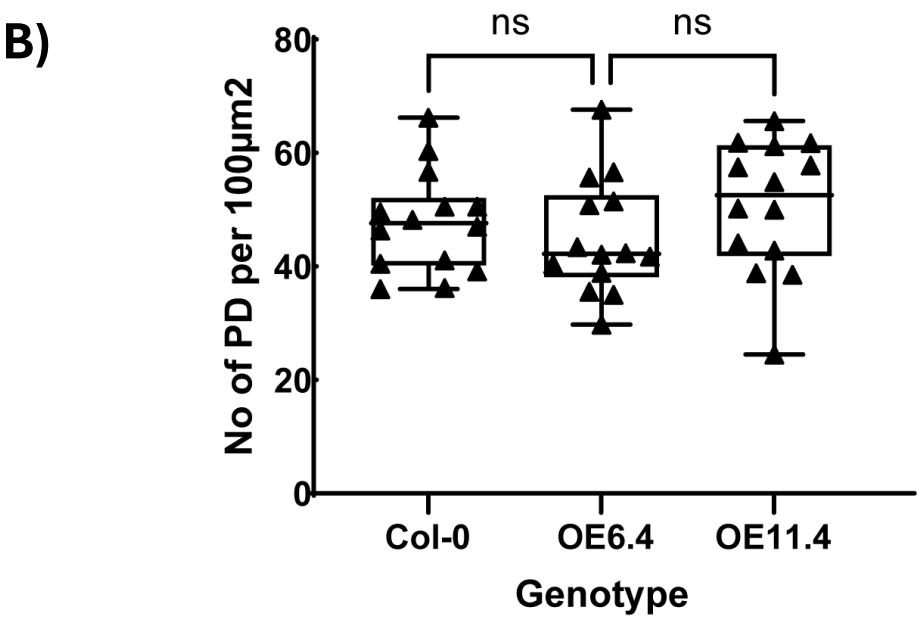

Supplementary Figure 3.

Supplement: Supplementary file 5 [file DataSheet2.pdf]
